# Supplementary material for: Fluoropyrimidine combination therapy versus fluoropyrimidine monotherapy for gemcitabine-refractory advanced pancreatic cancer: A systematic review and meta-analysis of randomized controlled trials
Source: PLoS One. 2023 Mar 2;18(3):e0282360. doi: 10.1371/journal.pone.0282360 (PMC9980826; doi:10.1371/journal.pone.0282360)
Supplement: S1 Appendix — (DOC) [file pone.0282360.s002.doc]

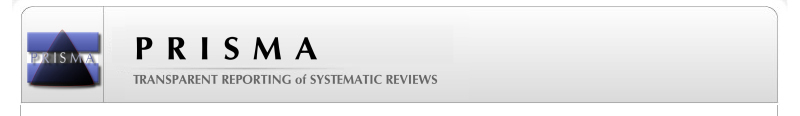
**PRISMA 2009 Flow Diagram**

**Screening**

**Included**

**Eligibility**

**Identification**

Records identified through database searching
(n = 1637)

Additional records identified through other sources
(n = 0)

Records after duplicates removed
(n = 1348)

Records screened
(n =1348)

Records excluded
(n = 1266)

315 reviews

618 for other drugs

298 for radiation or chemoradiation

35 case reports or others

Full-text articles assessed for eligibility
(n = 82)

Full-text articles excluded with reasons
(n = 76)

48 phase I trials

22 not-controlled design

6 not gemcitabine-based

Studies included in qualitative synthesis
(n = 6)

Studies included in quantitative synthesis (meta-analysis)
(n = 6)
